# Effects of X-ray on the metacestodes of *Echinococcus granulosus* in vitro

**DOI:** 10.1186/s12879-017-2741-x

**Published:** 2017-09-21

**Authors:** Rui Mao, Ge Wu, Hui Wang, Pengfei Lu, Jun Li, Haitao Li, Aimudula Ainiwaer, Yiwei Bai, Mingyang Shu, Yongxing Bao, Wenbao Zhang

**Affiliations:** 1 Department of Radiation Oncology, The First Affiliated Hospital of Xinjiang Medical University, Urumqi, 830054 China; 2 State Key Laboratory Incubation Base for Xinjiang Major Diseases Research and Xinjiang Key Laboratory of Echinococcosis, The First Affiliated Hospital of Xinjiang Medical University, Urumqi, 830054 China

**Keywords:** X-ray, *Echinococcus granulosus*, Metacestode, Infection

## Abstract

**Background:**

Radiotherapy may represent an alternative treatment modality for cystic echinococcosis (CE), but there is no adequate evidence for it up to now. In this study, we aim to investigate the parasiticidal effects of X-ray on the metacestodes of *Echinococcus granulosus* in vitro.

**Methods:**

Protoscoleces obtained from sheep naturally infected with CE were cultivated in RPMI 1640 medium containing 10% fetal bovine serum (FBS) at 37 °C in 5% CO_2_. Upon encystation on day 14, the metacestodes were subjected to various intensities of X-ray. Metacestode structures were observed using light microscope and transmission electron microscopy (TEM), and Real-Time PCR was carried out to determine the expression of *EgTPX*, *EgHSP70*, *EgEPC1* and *Caspase-3*.

**Results:**

On day 14, encystation was noticed in the majority of protoscoleces in the control group. In the X-ray groups, the encystation rate showed significant decrease compared with that of the control group (*P* < 0.05), especially the groups subjected to a dose of ≥40 Gy (*P* < 0.01). Light microscope findings indicated the hooklets on the rostellum were deranged in the irradiation group, and malformation was noticed in the suckers in a dose dependent manner. For the TEM findings, the cellular structure of the germinal layer of the cysts was completely interrupted by X-ray on day 7. The expression of *EgTPX*, *EgHSP70*, *EgEPC1* and *Caspase-3* was up-regulated after irradiation, especially at a dose of ≥45Gy (*P* < 0.05).

**Conclusions:**

X-ray showed parasiticidal effects on the metacestodes of *E. granulosus*. Irradiation triggered increased expression of *EgTPX*, *EgHSP70*, *EgEPC1* and *Caspase-3*.

## Background

Cystic echinococcosis (CE), a chronic and neglected disease with liver as the most frequent location of parasitic cysts [1], is a zoonosis induced by the larval stage of the cestode Echinococcus granulosus. The liver is the most frequent location of echinococcal cysts, accounting for about 70% of the cases, and the lungs are the second most common location [2]. However, the cysts can be detected anywhere in the whole body, and the clinical symptoms of the hydatid disease are highly depending on the size and sites of the lesions [3]. Surgery is preferred for treatment of CE. Nevertheless, severe anaphylactic shock may present in some patients due to postoperative release of fluid from the cysts. Besides, severe complications (e.g. secondary infection, cyst infection and hydrops) may result from leakage of CE cysts [4]. Albendazole (ABZ), as a chemotherapeutic agent used for the treatment of CE, has been commonly utilized in clinical practice, but its efficiency is hampered due to its disadvantages including poor absorption in gastrointestinal tract, poor solubility in water and organic solvents, as well as low concentrations in the parasite cysts [5]. Moreover, poor response was reported after medication of patients with hydatid cysts in the liver and lung, particularly those with bone hydatid disease. Therefore, it is necessary to develop new methods for the treatment of CE.

Radiotherapy may represent an alternative treatment modality for CE as it may induce cellular apoptosis or necrosis of CE, but up to now, there is no adequate evidence for it [6]. Only a few studies have been reported on the efficiency of radiotherapy in treating CE and the use of radiotherapy for the treatment of CE is still controversially discussed [7–9]. Besides, there are still disputes on the efficiency of radiotherapy on the treatment of CE infection. For example, in 1998, Schimid reported decrease of cyst in size after treatment using gamma knife radiosurgery in a case with cerebral alveolar hydatid disease [7]. In 2013, Ulger et al. revealed stable disease and absence of chest pain in one case who had failed the surgical procedures and medical treatment at the 1-year follow up after radiotherapy [8]. On the contrary, Pohle et al. concluded that radiotherapy should not be recommended for the management of alveolar echinococcosis as it had no clear-cut parasiticidal effects [9]. In 2013, radiotherapy using heavy ions was reported to promote the apoptosis of hydatid cysts through modulation of *Caspase 3* expression [10]. However, radiotherapy using heavy ions has been rarely performed in hospitals due to technical limitations. In this study, we investigated the parasiticidal effects of X-ray on the metacestodes of *E. granulosus* that mimicked the activities of *E. granulosus* in vivo. In addition, potential mechanisms were investigated.

## Methods

### Study setting and design

Fresh sheep liver samples infected with *E. granulosus* were used in the study. The sheep were obtained from a local sheep nursery in Xinjiang Autonomous Region. The animals were sacrificed according to the guide for the care and use of laboratory animals (8th edition). The study protocols were in line with the guidelines proposed by the First Affiliated Hospital of Xinjiang Medical University. Protoscoleces were subjected to irradiation, or they were cultured in vitro to investigate the effects of X-ray on the encystation.

### Culture of protoscoleces

The liver surface was washed with water, followed by sterilization using 75% ethanol. The hydatid fluid containing protoscoleces were obtained under aseptic conditions. Protoscoleces were washed using PBS (pH 7.2) for three times, followed by staining with 0.1% eosin for 5 min as previously described [11]. Then the staining results were observed under a light microscope. Dead protoscoleces stained in red color, and exhibited structural damages. Protoscoleces with a survival rate of >90% were used for the subsequent culture. The remaining protoscoleces were transferred to RPMI 1640 medium (HyClon, USA) containing 10% fetal bovine serum (HyClon, USA), and cultivated at 37 °C in 5% CO_2_. Encystation was noticed on day 10, and intact *E. granulosus* cyst were formed between day 14 and day 20.

### Effects of X-ray on the encystation of protoscoleces

Protoscoleces with a survival rate of >90% were divided into control group and X-ray groups subjected to X-ray with an intensity of 10 Gy, 20 Gy, 30 Gy, 40 Gy, 50 Gy, and 60 Gy [12], respectively. The viability was qualitatively determined using the eosin staining (0.1%) for 5 min, followed by observation using a microscope. The encystation rate was calculated based on the ratio of encystation to the total protoscoleces. The criteria of encystation were as follows: head enlargement, somites increase, integration of somites and hooklets, degradation of calcium granules, absence of rostellum [13]. The survival rate was calculated according to the number of protoscoleces underwent necrosis (staining in red color) in 100 protoscoleces. After cultivation for 14 days, the number of protoscoleces with encystation was observed under a microscope. The ratio of encystation was presented as the percentage of protoscoleces with encystation to the total number of protoscoleces.

### Effects of X-ray on the CE encystation

CE cysts were divided into four groups, including control group subject to no X-ray or X-ray groups subject to X-ray with an intensity of 30 Gy, 45 Gy and 60 Gy, respectively. In each group, three culture flasks containing a total of 600 cysts (200 cysts in each culture flask) were subjected to an irradiation dosage of 300 cGy/min at least three times. The distance between the X-ray source and the cyst was 100 cm. The irradiation field was 10 × 10 cm. The irradiation intensity was selected according to a dose escalation regimen, and the maximal intensity was selected to be 60 Gy that had been confirmed to be safety in human beings. The time interval of the 3 radiation cycle was 2 days, and was accomplished within 1 week. The radiation cycle and irradiation intensity were established according to the frequency and dosage of regimen used for treating liver cancer in clinical practice.

### Microscopic observation

On day 7, 100 cysts obtained from each group were observed under a light microscope to observe the morphology of the cysts. The protoscoleces were put onto slides, covered with coverslips, and observed microscopically under various magnifications.

### Transmission electron microscopy (TEM)

Protoscoleces were rinsed once with saline, fixed in 4% glutaraldehyde (24 h) and 1% osmium tetroxide sequentially, dehydrated with acetone gradient, and embedded in Epon 812 epoxy resin. The sections (60 nm) were cut with an ultrathin section machine, stained with uranium and lead electron stains, and observed under the TEM (JEOL1230, Japan).

### Real-time PCR

Total RNA was extracted from the cysts using TRIzol according to manufacturer’s instructions (Invitrogen, USA). The cDNA synthesis was carried out with approximately 2 μg RNA using the RevertAid First Strand cDNA Synthesis Kit (Thermo Fisher, USA). Real-time PCR was conducted using SYBR Green (QiaGen, Germany) on a Bio-Rad 785BR10642 system (Singapore) with the primers listed in Table 1, according to the manufacturer’s instructions. The PCR amplifications were as follows: 95 °C for 14 min, followed by 35 cycles of 95 °C 20 s, various annealing temperatures (*EgEPC1*: 55 °C; Caspase-3: 53 °C; *EgTPX*: 56 °C; *EgHSP70*: 56 °C) 30 s, and 72 °C 30 s, as well as 65 °C for 10 min. The mRNA level was normalized by beta-actin. The amplification results for real-time PCR was calculated as 2^(−ΔΔCt)^, according to the previous description [14].

**Table 1:** Specific primers of *EgTPX*, *EgEPC1* and *EgHSP70*

Gene	Primers (5′-3′)	Product size
*EgTPX*	F: TTTCTTAGATAAGCTCGACTCCAA R: AGTATATAGACCGGTGAATTAAGGG	196 bp
*EgHSP70*	F: GAGGAGTTGTGTTCGGACCT R: GTCCGGGTTTATCGACTTGT	145 bp
*EgEPC1*	F: TTTCTTAGATAAGCTCGACTCCAA R: AGTATATAGACCGGTGAATTAAGGG	151 bp
*Actin*	F: TCAATCCTAAAGCCAATC R: CGTACAACGACAGCAC	163 bp

### Statistical analysis

Data were analyzed by SPSS 17 0. Each test was performed at least in triplicate. Analysis of variance (ANOVA) was used for inter-group comparison. *P* < 0.05 was considered statistically significant.

## Results

### Effects of X-ray exposure on the encystation rate of protoscoleces

**Fig. 1:**
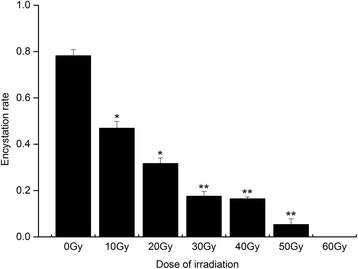
Encystation rate of protoscoleces after irradiation. ^*^ *P* < 0.05 versus control group; ^**^ *P* < 0.01 versus control group

On day 14, encystation was noticed in the majority of protoscoleces in the control group. Meanwhile, presence of the corneum layer was noticed in the peripheral part of the cyst. In the X-ray groups, the encystation rate was significantly decreased compared to the control group (*P* < 0.05), especially the groups subjected to a dose of 40 Gy or more (*P* < 0.01, Fig. 1). Radiation with an intensity of 60 Gy could completely inhibit the encystation.

### Morphology of protoscoleces after irradiation

**Fig. 2:**
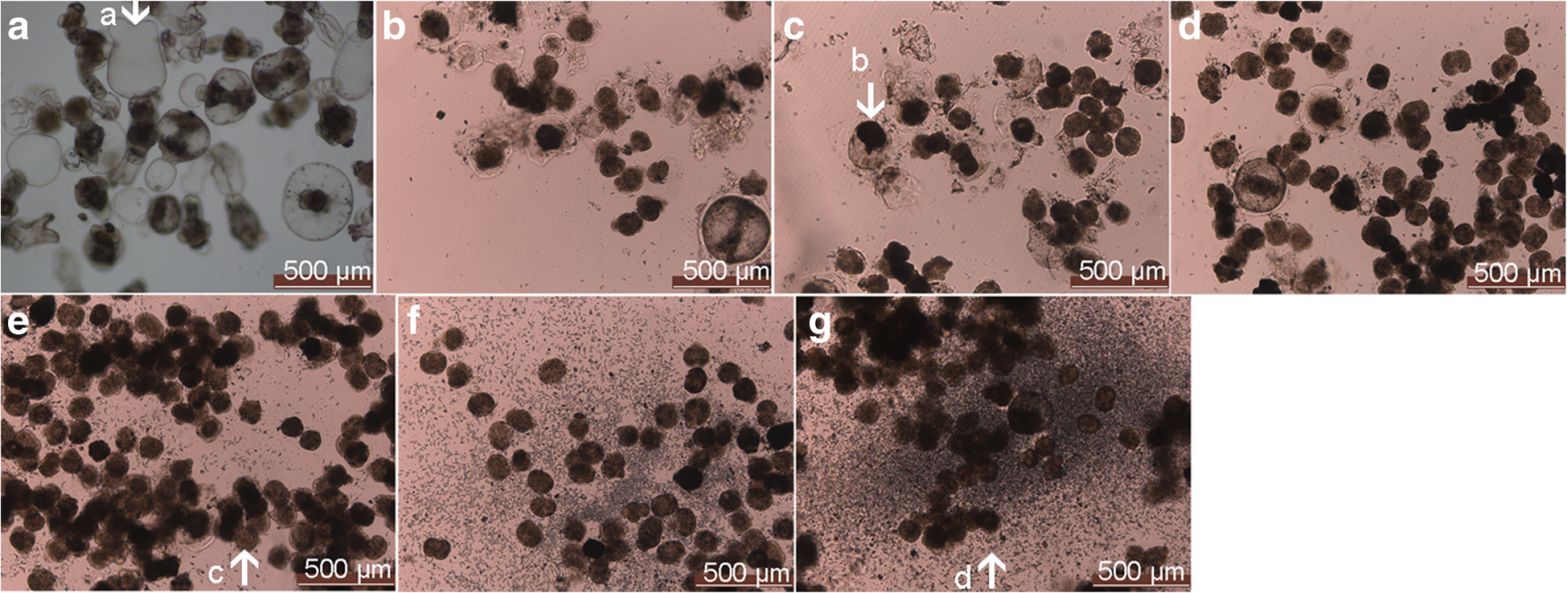
Morphologies of protoscoleces on day 14 after irradiation with a intensity of 0 Gy (**a**), 10 Gy (**b**), 20 Gy (**c**), 30 Gy (**d**), 40 Gy (**e**), 50 Gy (**f**), and 60 Gy (**g**). The protoscoleces were well developed in the control group, while interruption of hooklets was noticed in the irradiation group in a dose dependent manner. The images were observed under a magnification of 100×. a: normally developed and motile protoscoleces; b: non-motile protoscoleces; c: protoscoleces subject to necrosis; d: breakdown hooks

Compared with the control group, the structure of the protoscoleces was interrupted after X-ray exposure. In the control group, the protoscoleces were well developed with a higher encystation rate. The corneum layer could be observed around the cyst. The arrangement of hooklets on the rostellum was regular. In contrast, in the irradiation group, the hooklets on the rostellum were deranged, and malformation was noticed in the suckers in a dose dependent manner (Fig. 2).

### X-ray induced cystic wall atrophy

**Fig. 3:**
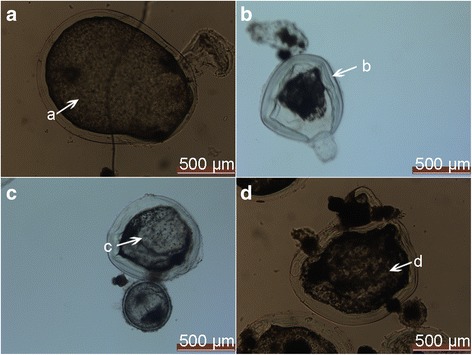
Morphologies of cyst after irradiation with a intensity of 0 Gy (**a**), 30 Gy (**b**), 45 Gy (**c**), and 60 Gy (**d**). The images were observed on day 7 under a magnification of 100×. a: calcium granules; b: collapsed cyst wall; c: elimination of calcium granules; d: cellular debris

In this part, we determined the growth of hydatids 1 week after X-ray exposure using light microscope. In the control group, the cyst wall was intact (Fig. 3a). On the contrary, in the X-ray groups, atrophy was noticed in the cyst walls. In addition, the structure of the cyst was completely disrupted after X-ray exposure (Fig. 3b-d).

### TEM findings

**Fig. 4:**
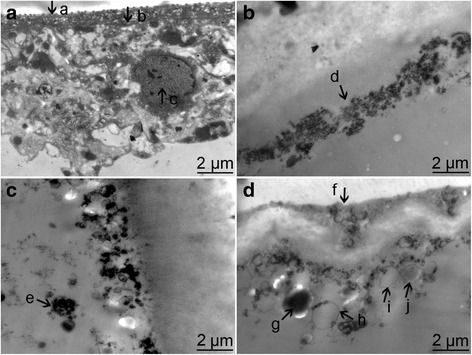
Morphology of cyst after irradiation under TEM. **a** The cystic structure was normal in the normal control. **b**-**d**: Cystic structures after irradiation under a density of 30 Gy, 45 Gy and 60 Gy. a: microtriches; b: tegument; c: undifferentiated cells; d. broken germinal layer; e: aberrantly condensed chromosomal masses; f: broken tegument; g: lipid droplets; h-j: empty vesicles

In the control group, the arrangement of the hydatid cysts was regular. The morphologies of cortex cells were intact. The nuclear structure was tight, and the nucleolus was clear. Meanwhile, electron dense bodies were dispersed in the cysts. However, after X-ray exposure, the structures were interrupted, together with dilatation of endoplasmic reticulum. Necrosis was noticed in the cortical cells, together with broken nuclei and disappearance of nucleoli (Fig. 4b). In addition, the cortical area was thin, and the microvilli were decreased in number (Fig. 4c). Particularly, in the 60 Gy group, swelling of mitochondria was noticed in the mitochondria in the cortical cells, combined with increase of lysosome and formation of myeloid body (Fig. 4d).

### Expression of *EgTPX*, *EgHSP70*, *EgEPC1* and *Caspase-3* in cyst after irradiation

**Fig. 5:**
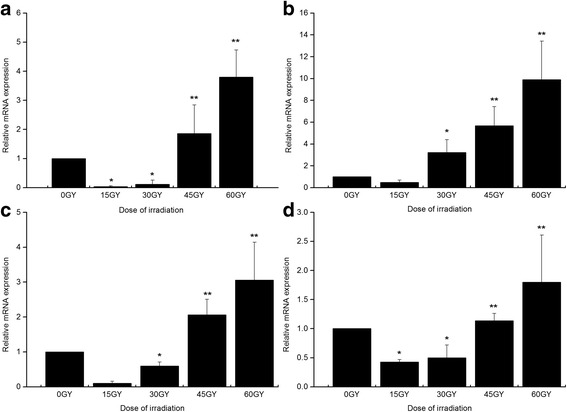
Expression of *EgTPX* (**a**), *EgHSP70* (**b**), *EgEPC1* (**c**) and *Caspase-3* (**d**) in cyst after irradiation in hydatid cysts. Gene expression was determined using Real-Time PCR. All the tests were performed at least in triplicate. ^*^ *P* < 0.05 versus control group; ^**^ *P* < 0.01 versus control group

The expression of *EgTPX* was down-regulated in the cyst after irradiation with a dose of 15 Gy and 30 Gy compared with the normal control. However, significant increase was noticed in the expression of *EgTPX* at a dose of 45 Gy and 60 Gy (Fig. 5a). The expression of *EgHSP70* was down-regulated after irradiation with a dose of 15 Gy compared with the control group. Nevertheless, its expression was significantly up-regulated after irradiation with a dose of 30 Gy, 45 Gy and 60 Gy compared with the control (Fig. 5b). For the expression of *EgEPC1* and *Caspase-3*, a remarkable down-regulation was noticed after a dose of 15 Gy or 30 Gy compared with the control group, but the expression was significantly up-regulated after a dose of 45 Gy or 60 Gy, respectively (Fig. 5c and d).

## Discussion

Previously, extensive studies have been carried out to investigate the efficiency of radiotherapy on *E. granulosus* protoscoleces [15, 16]. However, few studies have been carried out to investigate the efficiency of irradiation on metacestodes [9, 10]. In this study, we reported the effects of X-ray on in vitro cultured *E. granulosus* protoscoleces and the development of metacestodes, as well as already formed metacestodes. In addition, our study showed that irradiation showed parasiticidal effects through promoting the apoptosis in vitro.

Radiotherapy has been commonly used for the management of patients with cancer. Indeed, there are some side effects for radiotherapy such as percutaneous reaction, mucous reaction and bone marrow depression [17]. In this study, the irradiation dose was selected based on the tolerance of the tissues, in order to provide the rational for the potential application in vivo in future studies. To our best knowledge, only a few studies have been carried out to investigate the efficiency of radiotherapy on the management of CE [6]. Previously, Schmid reported a case of cerebral alveolar hydatid disease showed decrease of cyst diameter after treating with gamma knife radiosurgery and albendazole. The patient was followed up for at least 3 years with no nervous system symptoms [7]. In 2003, Park et al. investigated the survival of metacercariae irradiated with various amounts of gamma knife (5 Gy - 50 Gy) [18]. Compared with those of non-irradiated controls, recovery rates of adult worms in irradiated groups were reduced gradually with increased irradiation doses. No worm was recovered from rats fed with 50 Gy irradiated metacercariae. In this study, the structure of the protoscoleces was interrupted after X-ray exposure compared with the control group. To confirm the parasiticidal effects of X-ray on cysts, the cultured cysts in vitro were subject to irradiation, and the changes of the structures were observed using light microscope and TEM, respectively. Light microscope findings indicated presence of atrophy and collapse in the cyst wall, while in the control group, the wall was intact with no interruption. TEM showed dilatation of endoplasmic reticulum and aberrant condensed mass, which represented structural damages after irradiation, however, in the control group, the cysts were regularly arranged. On this basis, X-ray could interrupt the structure of hydatid cysts and induce apoptosis or necrosis.

Few studies have been carried out to investigate the molecular mechanism of irradiation on the hydatid cysts, and most of the studies have focused on the apoptosis [11]. For example, in a previous study, Yuan et al. revealed gamma knife induced higher mortality and up-regulation of *Caspase-3* in the protoscoleces [19]. Besides, Zhou et al. reported that heavy ions could promote the apoptosis of hydatid cysts through modulation of the expression of *Caspase 3* [10]. In order to investigate the potential mechanism in the irradiation on the hydatid cysts, we determined the expression of 3 genes that have been reported to be closely related to the growth of hydatids (i.e. *EgTPX*, *EgEPC1* and *EgHSP70*) and the caspase gene related to cellular apoptosis. The *EgTPX* gene was reported to involve in the clearance of reactive oxygen [20]. In this study, our results indicated that the expression of *EgTPX* was decreased with the increase of irradiation between 15 Gy and 30 Gy. On the contrary, its expression was increased with the increase of irradiation dose between 45 Gy and 60 Gy. On this basis, it is reasonable to conclude that a dose of 15–30 Gy inhibited the clearance capacity of hydatids to the oxygen radicals, while a dose of 45–60 Gy contributed to the clearance of oxygen radicals. For the expression of *EgHSP70* with anti-oxidant capacities closely involved in the protein translation [21], transfer and degradation, our results showed it was obviously increased upon irradiation with a dose of 30 Gy or more. With regards to the expression of *Caspase-3*, Hu et al. revealed *Caspase 3* involved in the apoptosis of protoscolex cells [11]. In this study, the expression of *Caspase 3* was decreased in a dose of 15–30 Gy, while it increased upon irradiation of a dose of 45–60 Gy. This indicated that a dose of 45–60 could trigger the apoptosis. Interestingly, as an epitope gene of *E. granulosa*, the expression of *EgEPC1* was down-regulated in presence of irradiation of 45 Gy and 60 Gy. In future, further studies are needed to investigate the potential causes.

Generally, hydatid cyst is considered as the life origin of *E. granulosa*. To our best knowledge, the research and management of *E. granulosa* related infection is hampered due to lacking of in vitro cultivation of metacestodes. In this study, we successfully cultivated metacestodes in vitro from the protoscoleces obtained from naturally infected sheep. On this basis, for the first time, we investigated the killing effects of irradiation on the metacestodes.

## Conclusion

We firstly investigated the parasiticidal effects of X-ray on the metacestodes of *E. granulosa* in vitro. X-ray showed parasiticidal effects and could modulate the phenotype and gene expression of the metacestodes. For the mechanism, it may be associated with the interruption of structure and cellular apoptosis.
